# Correction: Giantin is required for intracellular N-terminal processing of type I procollagen

**DOI:** 10.1083/jcb.20200516606072021c

**Published:** 2021-06-09

**Authors:** Nicola L. Stevenson, Dylan J.M. Bergen, Yinhui Lu, M. Esther Prada-Sanchez, Karl E. Kadler, Chrissy L. Hammond, David J. Stephens

Vol. 220, No. 6 | 10.1083/jcb.202005166 | May 4, 2021

The initial version of this article included a reference, Seeling et al. (1994), that was retracted because it was a duplicate publication. This reference has been replaced with the original article as follows:

Seelig, H.P., P. Schranz, H. Schröter, C. Wiemann, and M. Renz. 1994. Macrogolgin—a new 376 kD Golgi complex outer membrane protein as target of antibodies in patients with rheumatic diseases and HIV infections. *J. Autoimmun.* 7:67–91. https://doi.org/10.1006/jaut.1994.1006.

This change does not affect the study or its conclusions. The error appears only in print and in PDF versions downloaded on or before June 7, 2021.

